# Patients with a Short Distance Between the Prostate and the Rectum Are Appropriate Candidates for Hydrogel Spacer Placement to Prevent Short-Term Rectal Hemorrhage After External-Beam Radiotherapy for Prostate Cancer

**DOI:** 10.3390/curroncol32070385

**Published:** 2025-07-03

**Authors:** Shunsuke Owa, Takeshi Sasaki, Akito Taniguchi, Kazuki Omori, Taketomo Nishikawa, Momoko Kato, Shinichiro Higashi, Yusuke Sugino, Yutaka Toyomasu, Akinori Takada, Kouhei Nishikawa, Yoshihito Nomoto, Takahiro Inoue

**Affiliations:** 1 Department of Nephro-Urologic Surgery and Andrology, Mie University Graduate School of Medicine, 2-174 Edobashi, Tsu 514-8507, Mie, Japan; yamato4314@gmail.com (S.O.); t-sasaki@med.mie-u.ac.jp (T.S.); taketomo308070@med.mie-u.ac.jp (T.N.); momokok@med.mie-u.ac.jp (M.K.); s-higashi@med.mie-u.ac.jp (S.H.); y-sugino@med.mie-u.ac.jp (Y.S.); kouheini@med.mie-u.ac.jp (K.N.); 2 Department of Radiology, Mie University Graduate School of Medicine, 2-174 Edobashi, Tsu 514-8507, Mie, Japan; a-taniguchi@med.mie-u.ac.jp (A.T.); k-omori@med.mie-u.ac.jp (K.O.); y-toyomasu@med.mie-u.ac.jp (Y.T.); a-takada@med.mie-u.ac.jp (A.T.); nomoto-y@med.mie-u.ac.jp (Y.N.)

**Keywords:** prostate cancer, radiation therapy, hydrogel spacer

## Abstract

Radiation therapy, including external-beam radiation therapy (EBRT) and brachytherapy, is curative for localized prostate cancer. Hydrogel spacer (HS) placement between the rectum and prostate is popular for reducing radiation-related complications. Criteria to identify patients who benefit from HS placement would be clinically valuable. In a retrospective analysis of 430 patients with localized prostate cancer treated between November 2010 and March 2023 with ≥2 years of follow-up, we evaluated the incidence of rectal hemorrhage and its association with the median distance at the midpoint between the prostate and the rectum (mDPR) on pretreatment MRI. Rectal hemorrhage occurred in 6% of HS cases and 18% of non-HS cases (*p* < 0.001). Among 268 patients who received EBRT (±brachytherapy), the incidence was 9% with HS and 30% without HS (*p* < 0.001). In non-HS cases, the rate in patients with mDPR ≤ 1.62 mm was higher than in those with mDPR > 1.62 mm (24% vs. 12%, respectively; *p* = 0.04). In patients with EBRT and mDPR ≤ 1.62 mm, HS significantly reduced hemorrhage (9% vs. 39%, respectively; *p* < 0.001). Multivariate analysis identified mDPR and HS as independent predictors of rectal hemorrhage (both *p* = 0.02). Thus, HS placement may be safely omitted in non-EBRT cases with mDPR ≥ 1.62 mm.

## Simple Summary

External-beam radiation therapy is commonly used to treat prostate cancer, but it can sometimes cause side effects such as bleeding in the rectum. To reduce this risk, a soft gel called a hydrogel spacer can be inserted between the prostate and rectum. However, not every patient needs this procedure. In our study, we found that, among patients receiving external radiation therapy, those whose prostate and rectum are very close together are more likely to experience rectal bleeding after treatment. For these patients, using a hydrogel spacer greatly reduced the risk of bleeding. This means that, by measuring the distance before treatment, doctors can identify which patients undergoing external radiation will benefit most from a hydrogel spacer. These findings can help improve the safety of prostate cancer treatment and guide future decisions in clinical care.

## 1. Introduction

The incidence and mortality rates of prostate cancer are increasing consistently in Japan [1], and radiation therapy, including external-beam radiation therapy (EBRT) and brachytherapy, is widely used as a treatment option for localized prostate cancer [2,3,4]. A hydrogel spacer (HS) is placed between the rectum and prostate during radiation therapy to maintain physical distance, thereby reducing the risk of radiation-related complications [5]. While HS placement is not technically difficult, it is not completely free of adverse events, such as rectal ulcers or infections [6,7]. Currently, there are no clear criteria for determining whether to retain HSs, and the decision is left to the discretion of the attending physician. From a health economics perspective, it has not been proven that placement of HS is appropriate for all cases, and criteria for the use of HS placement must be established. However, few studies have addressed this issue. In particular, the midline distance between the prostate capsule and the rectal mucosa (DPR), measurable on pretreatment magnetic resonance imaging (MRI), may serve as a practical and non-invasive indicator to guide HS placement. This study aimed to retrospectively evaluate the association between DPR and the development of rectal hemorrhage—a gastrointestinal adverse event that can occur shortly after initiating radiation therapy—in patients with prostate cancer. This approach has the potential to support appropriate HS placement decisions without requiring additional procedures or testing.

## 2. Patients and Methods

This study was approved by the Ethics Committee of the Affiliated Hospital of the Faculty of Medicine, Mie University (Approval Number: H2020-195). This was a retrospective analysis of 430 cases among 499 patients who underwent radiation therapy for localized prostate cancer at the Affiliated Hospital of the Faculty of Medicine, Mie University, between November 2010 and March 2023 with ≥2 years of follow-up. Patients with metastasis at the time of diagnosis or those who received chemotherapy prior to radiation therapy were excluded.

Of the 430 registered patients, 204 were in the HS implantation group and 226 were in the non-implantation group. HS implantation has been performed at our hospital since February 2019 in all cases except those in which prostate cancer was located on the rectal side and deemed unsuitable for implantation. Table 1 shows the patient background characteristics for each group. In the EBRT group, treatment was performed using intensity-modulated radiation therapy (IMRT), and all brachytherapy was performed using iodine-125 seeds. In the brachytherapy-alone group, radioactive seed implantation was performed first, followed by hydrogel spacer (HS) placement during the same procedure. In the combined brachytherapy and EBRT group, the same approach was used—brachytherapy and HS placement were performed on the same day, and EBRT was initiated approximately one month later. In our protocol, HS was placed after brachytherapy but before EBRT. This sequence was necessary because brachytherapy was performed under transrectal ultrasound guidance, and placing the HS beforehand would have interfered with ultrasound visualization of the prostate, making accurate seed implantation technically difficult. Accordingly, the radiation dose in the HS implantation group was that evaluated before HS implantation for brachytherapy and that evaluated after HS implantation for EBRT. Table 2 shows the treatment strategies. The indications for EBRT and brachytherapy were determined primarily by the National Comprehensive Cancer Network (NCCN) risk classification. In addition, patients were instructed to evacuate gas and their bowels prior to EBRT to prevent rectal adverse events, and laxatives were prescribed as needed. In our institution, patients treated with EBRT alone received a total dose of 74 Gy in 37 fractions (2 Gy per fraction), whereas those who underwent combined brachytherapy and EBRT received 45 Gy in 20 fractions (2.25 Gy per fraction) for the EBRT component. Accordingly, based on the differences in prescribed EBRT dose, rectal dose evaluation was performed using rectal V70 for the EBRT-only group and rectal V40 for the combined-modality group. All patients included in this study were treated with conventional fractionation regimens prior to July 2024, at which point our institution implemented a moderately hypo-fractionated protocol based on the CHHiP trial. Thus, no patients receiving moderately hypo-fractionated or stereotactic body radiotherapy (SBRT) were included in the present analysis. In our current clinical practice, for patients with pelvic lymph node metastases (N1), we employ a simultaneous integrated boost (SIB) approach to treat both the primary prostate lesion and the pelvic nodal regions. However, as N1 cases were excluded from this study, our findings do not directly pertain to these populations.

From February 2020 onward, our institution transitioned from static-field IMRT (sliding window) to volumetric modulated arc therapy (VMAT) as the standard external beam radiotherapy (EBRT) technique. Consequently, the majority of patients treated after this date received VMAT. To assess the potential impact of this change on rectal toxicity, we conducted subgroup analyses comparing patients treated before and after February 2020, stratified by hydrogel spacer (HS) placement status. Pretreatment verification was performed using both kilovoltage (kV) imaging and cone-beam computed tomography (CBCT). While CBCT is considered the most accurate method for setup verification, its routine use has certain drawbacks, including increased radiation exposure and longer treatment times. Therefore, our institution adopted kV–kV imaging as the standard daily setup method. During the first three treatment sessions, both CBCT and kV–kV imaging were performed, and if the alignment between the two modalities was consistent, then subsequent sessions were conducted using kV–kV imaging alone. In cases where discrepancies were identified, the frequency of CBCT imaging was appropriately increased to minimize prostate displacement caused by rectal contents. Furthermore, Visicoil fiducial markers were implanted in all patients undergoing EBRT to facilitate accurate daily localization and further reduce the risk of target misalignment. Daily image-guided radiotherapy (IGRT) was routinely performed for all patients undergoing EBRT for prostate cancer.

Adverse events associated with radiation therapy were analyzed by evaluating the incidence of rectal hemorrhage within 2 years after the start of treatment. Endoscopic evaluation of rectal hemorrhage was not performed in all cases. Complications were evaluated based on the Common Terminology Criteria for Adverse Events (CTCAE) Version 5.0 (CTCAE Ver 5.0), and the number of patients in each grade was counted [8].

**Figure 1:**
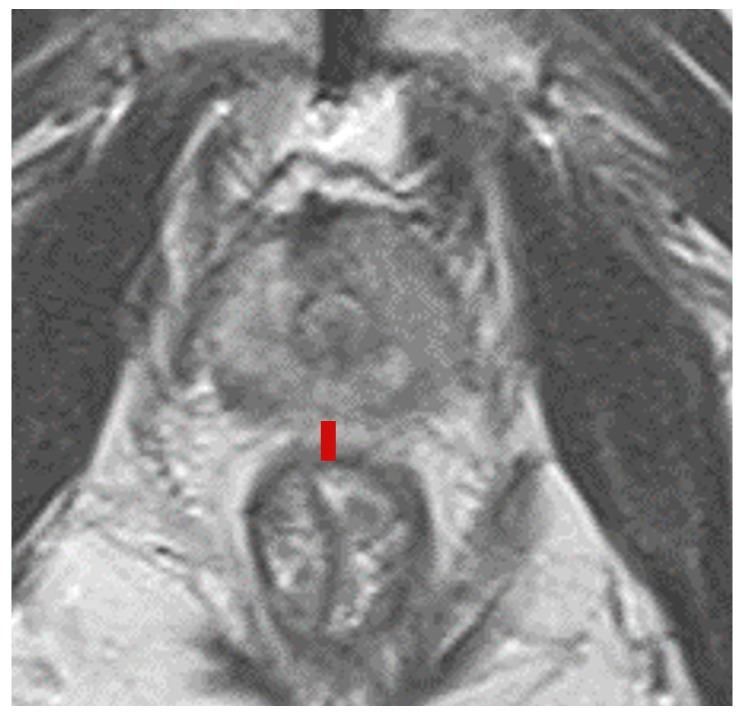
The distance from the prostate capsule to the rectal mucosa (DPR) was measured using horizontal MRI images obtained before prostate biopsy or treatment. DPR was measured at three locations: the apex, midpoint, and base of the prostate. The median value of the DPR measured at the midpoint was defined as the mDPR. The red line indicates the DPR.

First, the incidence of hemorrhage was compared between the HS implantation group and the non-HS implantation group, and a chi-square test was performed for each item. Second, the occurrence of rectal hemorrhage was evaluated by a chi-square test according to treatment. Overall radiation therapy was divided into three groups: EBRT treatment, brachytherapy alone, and brachytherapy + EBRT (including trimodal). Third, the DPR was evaluated using pretreatment MRI horizontal images (Figure 1). All distance measurements were performed by a single evaluator. This distance was measured at the prostate apex, midpoint (exactly halfway between the apex and the base), and the base. The MRI images used to measure the distance between the prostate and rectum (DPR) were taken prior to the initiation of both hormone therapy and radiotherapy. The median DPR for each location was calculated, and the relationship with the incidence of rectal hemorrhage was evaluated using Fisher′s exact test. Next, univariate and multivariate regression analyses were performed to identify factors associated with the occurrence of rectal hemorrhage. In the univariate analysis, the relationship between each factor and rectal hemorrhage was evaluated individually, and then multiple factors were adjusted simultaneously using multivariate regression analysis. For statistical analysis, EZR (Saitama Medical Center, Jichi Medical University, Japan), an interface for R (R Foundation for Statistical Computing, Vienna, Austria) was used [9].

## 3. Results

**Figure 2:**
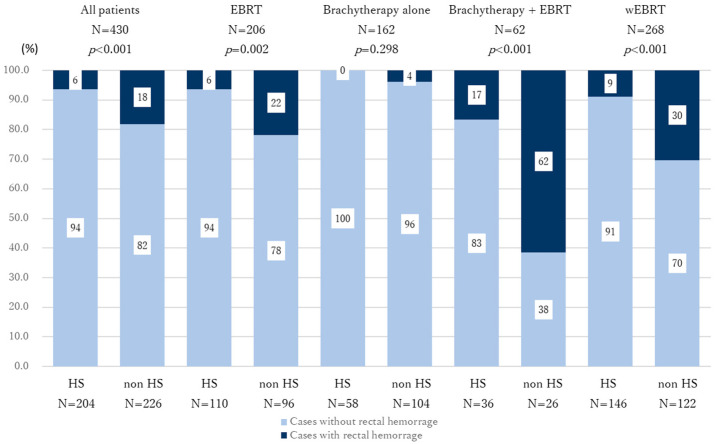
Graph showing the incidence of rectal hemorrhage stratified with or without HS placement. The numbers in the graph indicate the percentage within each group. A significant difference in rectal hemorrhage incidence between the HS and non-HS groups was observed in the EBRT group, the brachytherapy + EBRT group, and the wEBRT group. In contrast, no significant difference was observed in the brachytherapy-alone group. Dark blue: cases with rectal bleeding; faint blue: cases without rectal bleeding.

First, we analyzed the incidence of rectal hemorrhage of all grades between the HS implantation group and the non-HS implantation group among all cases (*n* = 204 vs. *n* = 226). A significantly lower incidence of rectal hemorrhage was observed in the HS group compared with the non-HS group (*p* < 0.001). Grade 3 rectal hemorrhage occurred in only two patients, both in the non-HS group. Second, the incidence of rectal hemorrhage was compared between the two groups according to treatment modality. In the EBRT alone group, the incidence was 6% in the HS group (*n* = 110) and 22% in the non-HS group (*n* = 96) (*p* < 0.001). In the brachytherapy + EBRT combination group, rectal hemorrhage occurred in 16.7% of HS patients (*n* = 36) and 61.5% of non-HS patients (*n* = 26) (*p* < 0.001). However, in the brachytherapy-alone group, no significant difference was observed (HS group: 0%, *n* = 58; non-HS group: 4%, *n* = 104; *p* = 0.298) (Figure 2). In addition, when EBRT-treated patients were grouped together (wEBRT: EBRT alone and brachytherapy + EBRT), the rectal hemorrhage incidence was 9% in the HS group (*n* = 146) and 30% in the non-HS group (*n* = 122), representing a significant difference (*p* < 0.001) (Figure 2). Subgroup analyses were performed in the EBRT group to evaluate the impact of the transition to VMAT (February 2020) on rectal bleeding. Before the transition, 112 patients were treated (HS: 23, non-HS: 89); after the transition, 93 patients were treated (HS: 87, non-HS: 6). Rectal bleeding occurred in 0.0% (0/23) before and 8.0% (7/87) after the transition in the HS group, and in 22.5% (20/89) before and 16.7% (1/6) after in the non-HS group (*p* = 0.341 and *p* = 1.00, respectively).

**Figure 3:**
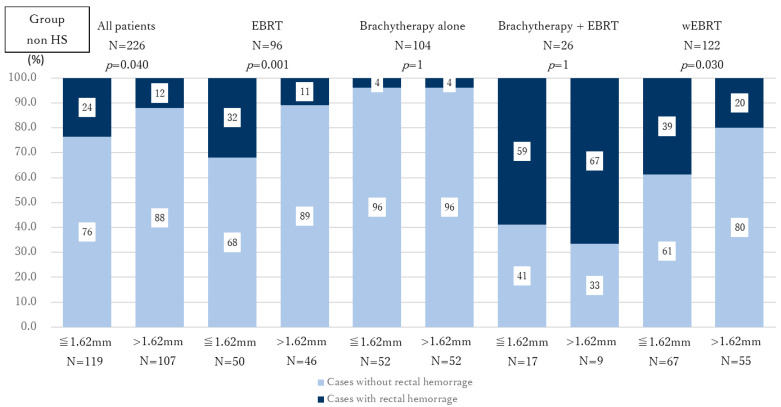
Comparison of rectal hemorrhage incidence according to mDPR among non-HS patients. The numbers in the graph indicate the percentage within each group. In the overall cohort, EBRT group, and wEBRT group, patients with mDPR ≤ 1.62 mm had a significantly higher incidence of rectal hemorrhage than those with mDPR > 1.62 mm. Dark blue: cases with rectal bleeding; faint blue: cases without rectal bleeding.

**Figure 4:**
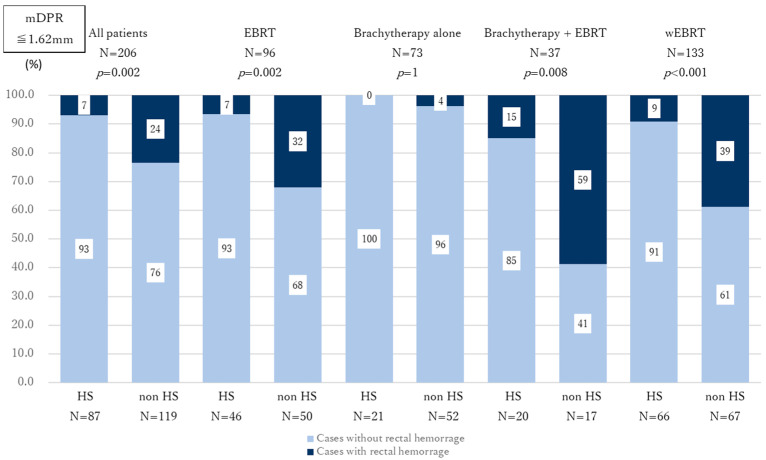
Effect of HS placement on the incidence of rectal hemorrhage among patients with mDPR ≤ 1.62 mm. HS placement significantly reduced the incidence of rectal hemorrhage in patients undergoing EBRT with mDPR ≤ 1.62 mm. Dark blue: cases with rectal bleeding; faint blue: cases without rectal bleeding.

Third, among the 499 patients initially enrolled in this study, the DPR was measured using axial MRI images at the base, midline, and apex of the prostate in all cases. The median DPRs were 14.17 mm (IQR: 9.60–19.98) at the base, 1.62 mm (IQR: 1.16–2.28) at the midline, and 1.49 mm (IQR: 1.10–2.15) at the apex. When the patients in the non-HS group were stratified by the median value of midline DPR (mDPR = 1.62 mm), the incidence of rectal hemorrhage was significantly higher in those with mDPR ≤ 1.62 mm compared with those with mDPR > 1.62 mm in the overall cohort, the EBRT-alone group, and the wEBRT group (*p* = 0.040, *p* = 0.001, and *p* = 0.030, respectively; Figure 3; Supplementary Figure S1). No statistically significant differences in rectal hemorrhage incidence were observed when results were stratified by median DPR at the base or apex. Based on this finding, we compared the incidence of rectal hemorrhage between the HS and non-HS groups in patients with mDPR ≤ 1.62 mm. A significant reduction in rectal hemorrhage was observed in the HS group across the overall cohort, the EBRT-alone group, and the wEBRT group (*p* = 0.002, *p* = 0.002, and *p* < 0.001, respectively; Figure 4; Supplementary Figure S2).

Based on these results, we conducted univariate and multivariate analyses to identify factors associated with rectal hemorrhage in the EBRT-alone group, which included the largest number of patients. In the univariate analysis, significant associations were observed for mDPR, HS implantation, and rectum V70 (%) (*p* = 0.02, *p* < 0.01, and *p* = 0.02, respectively). In the multivariate analysis, both mDPR and HS placement remained independently associated with the incidence of rectal hemorrhage (*p* = 0.02 and *p* = 0.02, respectively; Table 3). These findings suggest a significantly higher risk of rectal hemorrhage in patients with shorter mDPR and without HS placement.

## 4. Discussion

Radiation therapy is widely employed worldwide as a curative treatment for localized prostate cancer. When EBRT or brachytherapy is administered, the anatomical proximity of the prostate to the rectum raises concerns about radiation-induced rectal toxicity [10]. Although the rectum is fixed within the pelvis, its volume changes due to the accumulation of gas and feces, and it may enter the radiation field for the prostate, causing radiation exposure to the anterior wall of the rectum [11]. Various measures are taken to control rectal volume and stabilize pelvic anatomy, including rectal balloon placement, the administration of laxatives, dietary management, and the regulation of urination [12,13,14,15]. In addition, the use of topical medications during radiotherapy and the choice of radiation delivery technique have also been reported to influence the incidence of rectal bleeding. In this study, no prophylactic topical medications were used for the prevention of rectal bleeding among the enrolled patients [16]. To mitigate these complications, HSs are frequently placed between the prostate and rectum. Clinical trials have demonstrated that HS placement can reduce rectal radiation-related adverse events significantly [5]. Although HS placement is considered beneficial in minimizing rectal toxicity, it is not entirely without risk. Adverse events related to HS insertion, such as rectal ulcers and infections, have been reported [6,7]. While numerous studies have addressed the effectiveness of HSs in reducing radiation-related complications, few have focused on defining appropriate indications for its use. Selective HS placement in patients most likely to benefit could improve treatment efficiency and provide meaningful advantages from a health economics perspective.

As in previous studies, our results confirmed that HS implantation reduced the short-term incidence of rectal hemorrhage following radiation therapy across the entire cohort [17,18,19]. However, when stratified by treatment type, no significant reduction in rectal hemorrhage was observed in the brachytherapy-alone group. In brachytherapy, both the rectal volume receiving 100% of the prescribed dose (RV100) and the distance between the prostate and rectum have been reported as risk factors for rectal hemorrhage [20,21]. In addition, in cases where brachytherapy is combined with EBRT, RV100 and patient age have been identified as contributing factors [22]. In our analysis, however, no significant association was found between RV100 or age and the incidence of rectal hemorrhage in either the brachytherapy-alone group or the brachytherapy + EBRT group (*p* = 0.846 and *p* = 0.414, respectively). Numerous studies have also suggested a causal relationship between rectum V70 (%) and rectal hemorrhage in EBRT [23]. At our institution, the rectal margin is uniformly maintained during EBRT; however, irradiation of the seminal vesicles is modified based on NCCN risk classification. Specifically, no seminal vesicle irradiation is performed for patients with low risk; irradiation is extended 1 cm from the base of the seminal vesicles for intermediate risk; and for high risk, either 2 cm from the base or the entire seminal vesicles are irradiated. In the non-HS group, no significant difference in rectum V70Gy (%) was observed among the three risk groups (*p* = 0.539).

In this study, pretreatment axial MRI images were used to measure the distance between the prostate and the rectum. The use of contrast agents and differences in MRI field strength (1.5 T or 3.0 T) were not taken into consideration. While previous studies have typically used sagittal images to assess the prostate–rectum distance, sagittal MRI is not always available in all clinical settings [24]. Therefore, we evaluated the distance using axial prostate MRI images. Our findings demonstrated that the mDPR was associated with the incidence of rectal hemorrhage. The median mDPR in this study was 1.62 mm, which appears consistent with prior reports citing a median prostate–rectum distance of 1.62 ± 2.2 mm [5]. Although the anatomical distance is shortest at the prostate apex, only the midline measurement showed a significant correlation with rectal hemorrhage in our cohort. One possible explanation is that stool or gas accumulation within the rectum can cause distension of the rectal wall, which in turn displaces the prostate. This displacement may occur in the superior–internal or anteroposterior directions, particularly affecting the mid-gland region, where the rectal wall is more mobile compared to the apex [24,25,26,27]. Consequently, the mid-gland area may be more prone to unintended radiation exposure, even with accurate treatment planning. In addition, previous reports have suggested that the upper rectum may be more prone to radiation-induced rectal hemorrhage due to anatomical and functional differences compared with the lower rectum [28].

The results of this study demonstrated that rectal hemorrhage occurred more frequently in the non-HS group, the EBRT-alone group, and the wEBRT group when the mDPR was 1.62 mm or less. In these patient groups, the use of HS significantly reduced the incidence of rectal hemorrhage, confirming the preventive effect of HS placement. Although HS implantation did not lead to a reduction in rectal hemorrhage in the brachytherapy-alone group, this finding is consistent with previous studies that also failed to demonstrate a benefit of HS placement in the absence of EBRT [29]. Multivariate analysis limited to the EBRT group further confirmed that both mDPR and HS placement were independently associated with the occurrence of rectal hemorrhage (*p* = 0.02 for both). In contrast, rectum V70 (%) was not associated significantly with rectal hemorrhage in this analysis. This may be attributed to the relatively low rectal radiation dose at our institution compared with values reported in earlier studies, particularly at the time of HS placement [23].

This study has several limitations. First, it was retrospective in nature, and although endoscopic evaluation of rectal bleeding was performed in some cases, it was not conducted systematically in all patients. Therefore, the exact cause of rectal bleeding could not be definitively attributed to radiation-induced proctitis in every case. Second, differences in follow-up duration between the HS and non-HS groups prevented meaningful analysis of chronic or late-onset complications. Nonetheless, previous studies have suggested that patients who do not experience short-term adverse events are at a lower risk of developing late complications, although further research is warranted to evaluate long-term outcomes [30]. Third, in this study, DPR was assessed using MRI scans obtained prior to the initiation of hormone therapy. Although hormone therapy may influence prostate volume, no significant association was found between hormone therapy and rectal bleeding in our analysis (*p* = 0.779 for the overall cohort) [31,32]. Subgroup analyses by treatment modality also showed no significant associations (external-beam radiotherapy: *p* = 0.586; brachytherapy alone: *p* = 0.696; combined therapy: *p* = 0.536). Furthermore, as prostate volume—which is influenced by hormone therapy—did not show a significant association in either univariate or multivariate analyses, the impact of hormone therapy in this study is considered to be limited. Fourth, our analysis was limited to short-term adverse events. From the perspective of short-term toxicity, rectal-related adverse events following radiation therapy for localized prostate cancer have been reported to affect patient quality of life significantly. In this context, assessing the incidence of rectal hemorrhage within a two-year follow-up period provides meaningful insight into short-term safety outcomes [33]. However, we should extend the follow-up and evaluate long-term adverse events between the HS and non-HS groups.

The results of this study suggest that, for the purpose of preventing short-term rectal hemorrhage within two years, HS placement may be considered for patients receiving EBRT with an mDPR of 1.62 mm or less. However, rectal complications are multifactorial and influenced by various elements, including radiation delivery techniques, imaging modalities, dose planning strategies, patient-specific tolerance, and image guidance protocols [34,35,36,37]. We hope that our findings will be interpreted within the context of these contributing factors and will inform appropriate strategies for minimizing rectal toxicity [11]. In recent years, less invasive treatments such as thermal-energy-based ablation and focal therapy have gained attention in the management of prostate cancer [38]. These approaches are associated with reduced treatment-related toxicity and reflect a broader shift toward precision-based interventions. While our study does not focus on focal therapy, the use of anatomical measurements such as mDPR to guide HS placement aligns with this trend toward individualized treatment.

In future studies, we aim to conduct longer follow-up and evaluate other rectal-related adverse events beyond rectal hemorrhage.

## 5. Conclusions

When the mDPR is 1.62 mm or greater, HS placement may be safely omitted in non-EBRT cases; however, because rectal complications are influenced by multiple factors, including treatment planning and delivery methods, a comprehensive clinical assessment remains essential. Notably, this study evaluated short-term toxicity (rectal bleeding) within two years of treatment initiation and does not provide guidance for the prediction or prevention of late-onset rectal toxicity.

## Figures and Tables

**Table 1:** Patient characteristics.

	HS	Non HS
Number of patients	204	226
Median (IQR) age, years	72 (68–76)	71.5 (67–75)
Prostate volume, cc, (IQR)	24.0 (18.2–31.5)	26.9 (19.1–37.0)
Clinical T stage at diagnosis, *n* (%)		
T1/T2/T3a/T3b/T4	37/132/22/11/2 (18.1/64.7/10.8/5.4/1.0)	82/105/22/9/8 (36.3/46.5/9.7/4.0/3.5)
Biopsy grade group, *n* (%)		
1/2/3/4/5	68/50/38/24/24 (33.3/24.5/18.6/11.8/11.8)	100/49/33/25/19 (44.2/21.7/14.6/11.1/8.4)
NCCN risk group, *n* (%)		
Very low	4 (2.0)	9 (4.0)
Low	41 (20.1)	74 (32.7)
Favorable intermediate	41 (20.1)	50 (22.1)
Unfavorable intermediate	54 (26.5)	33 (14.6)
High	47 (23.0)	39 (17.3)
Very high	17 (8.3)	21 (9.3)
Radiation therapy type, *n* (%)		
EBRT (IMRT)	110 (53.9)	96 (42.5)
Brachytherapy alone	58 (28.4)	104 (46.0)
Brachytherapy + EBRT	29 (14.2)	22 (9.7)
Brachytherapy + EBRT + hormonal therapy	7 (3.4)	4 (1.8)
Dose evaluation, median		
EBRT (IMRT), % (IQR)		
EBRT rectum V70 Gy	0.00 (0.00–0.00)	0.69 (0.13–2.15)
EBRT bladder V70 Gy	8.43 (4.66–11.59)	15.25 (8.42–19.74)
Brachytherapy alone		
RV100, mL (IQR)	0.230 (0.103–0.425)	0.120 (0.038–0.263)
D90, % (IQR)	110.1 (107.0–112.8)	113.9 (109.4–117.6)
Brachytherapy + EBRT		
RV100, mL (IQR)	0.310 (0.170–0.560)	0.160 (0.025–0.338)
D90, % (IQR)	113.6 (109.1–117.1)	113.4 (110.2–116.9)
EBRT rectum V40 Gy, % (IQR)	0.01 (0.0–6.72)	19.64 (14.03–24.41)
EBRT bladder V40 Gy, % (IQR)	13.08 (8.19–16.99)	19.38 (13.22–26.41)
Brachytherapy + EBRT + hormonal therapy		
RV100, mL (IQR)	0.110 (0.060–0.160)	0.295 (0.198–0.405)
D90, % (IQR)	111.0 (108.9–112.4)	108.0 (107.5–110.7)
EBRT rectum V40 Gy, % (IQR)	0.00 (0.00–0.07)	49.86 (39.23–58.00)
EBRT bladder V40 Gy, % (IQR)	10.96 (7.40–12.27)	36.01 (29.99–44.26)

NCCN: National Comprehensive Cancer Network; EBRT: external-beam radiation therapy, IMRT: intensity-modulated radiation therapy; RV100: rectal volume receiving 100% of the prescribed dose; D90: dose covering 90% of the target volume; V70: volume of organ receiving 70 Gy.

**Table 2:** Treatment strategies.

EBRT (IMRT; 74 Gy/34 fr)	
Very low/low Favorable intermediate	EBRT only
Unfavorable intermediate	6 months to 1 year HT + EBRT
High/very high	6 months to 1 year HT + EBRT + 2-year HT
Brachytherapy	
Very low/low Favorable intermediate	Brachytherapy only
Unfavorable intermediate	Brachytherapy + EBRT (45 Gy/20 fr)
High/very high	6 months HT + brachytherapy + EBRT (45 Gy/20 fr) + 2 years HT

HT: hormonal therapy; EBRT: external-beam radiation therapy.

**Table 3:** Univariate/multivariate analysis of factors related to the incidence of rectal hemorrhage in the EBRT group.

Factor	Univariate			Multivariate		
	OR	95% CI	*p* Value	OR	95% CI	*p* Value
cT (≥cT3 vs. ≤cT2)	2.02	0.90–4.51	0.09	-	-	-
mDPR ≥ 1.62	0.36	0.16–0.84	0.02	0.36	0.15–0.88	0.02
HS vs. non-HS	0.24	0.10–0.60	<0.01	0.32	0.12–0.86	0.02
Rectum V70 Gy (%)	1.30	1.04–1.61	0.02	1.16	0.90–1.50	0.24
Gleason Grade (≥4 vs. ≤3)	1.70	0.76–3.78	0.20	-	-	-
PSA	1.01	1.00–1.01	0.09	-	-	-
NCCN risk	1.58	0.80–3.12	0.19	-	-	-
Prostate volume	1.01	0.98–1.03	0.53	-	-	-
Age	1.00	0.93–1.09	0.91	-	-	-

mDPR: the median value of the distance from the prostate capsule to the rectal mucosa at the midline; HS: hydrogel spacer; V70: volume of organ receiving 70 Gy; NCCN: National Comprehensive Cancer Network.

## Data Availability

The data presented in this study are not publicly available due to privacy and ethical restrictions.

## Supplementary Materials

The following supporting information can be downloaded at: https://www.mdpi.com/article/10.3390/curroncol32070385/s1, Figure S1: Comparison of rectal hemorrhage incidence according to mDPR among HS patients; Figure S2: Effect of HS placement on the incidence of rectal hemorrhage among patients with mDPR > 1.62 mm.

## Abbreviations

The following abbreviations are used in this manuscript:

HS	hydrogel spacer
EBRT	external-beam radiation therapy
DPR	distance between prostate and rectum
mDPR	median distance at midpoint between the prostate and rectum
IMRT	intensity-modulated radiation therapy
RV100	rectal volume receiving 100% of prescribed dose
V60 Gy/V70 Gy	rectal volume receiving ≥60 or ≥70 Gy
D90	dose received by 90% of the target volume
HT	hormonal therapy
