# Supplementary material for: Patients with a Short Distance Between the Prostate and the Rectum Are Appropriate Candidates for Hydrogel Spacer Placement to Prevent Short-Term Rectal Hemorrhage After External-Beam Radiotherapy for Prostate Cancer
Source: Curr Oncol. 2025 Jul 3;32(7):385. doi: 10.3390/curroncol32070385 (PMC12293744; doi:10.3390/curroncol32070385)
Supplement: Supplementary file 1 [file curroncol-32-00385-s001.zip › curroncol-3693131-supplementary.pdf]

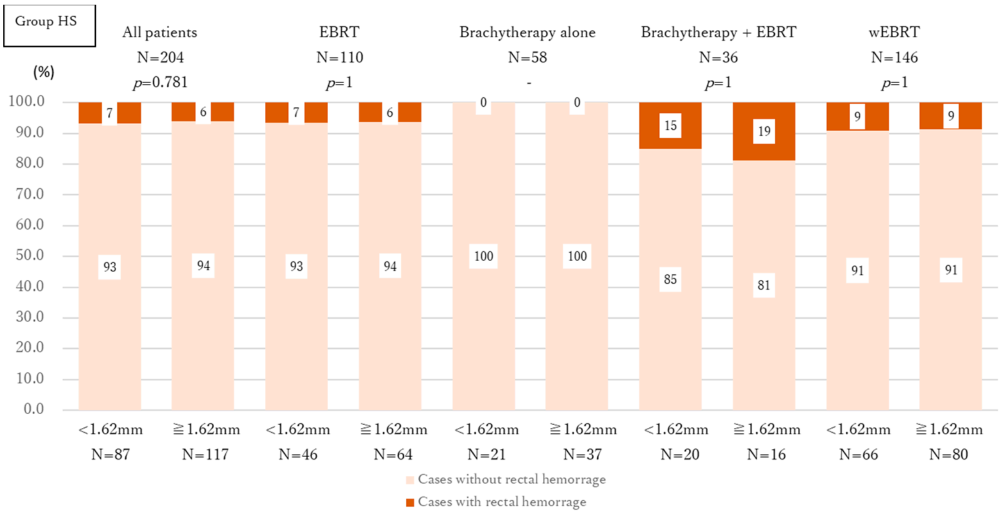


Figure S1: Comparison of rectal hemorrhage incidence according to the mDPR among HS patients

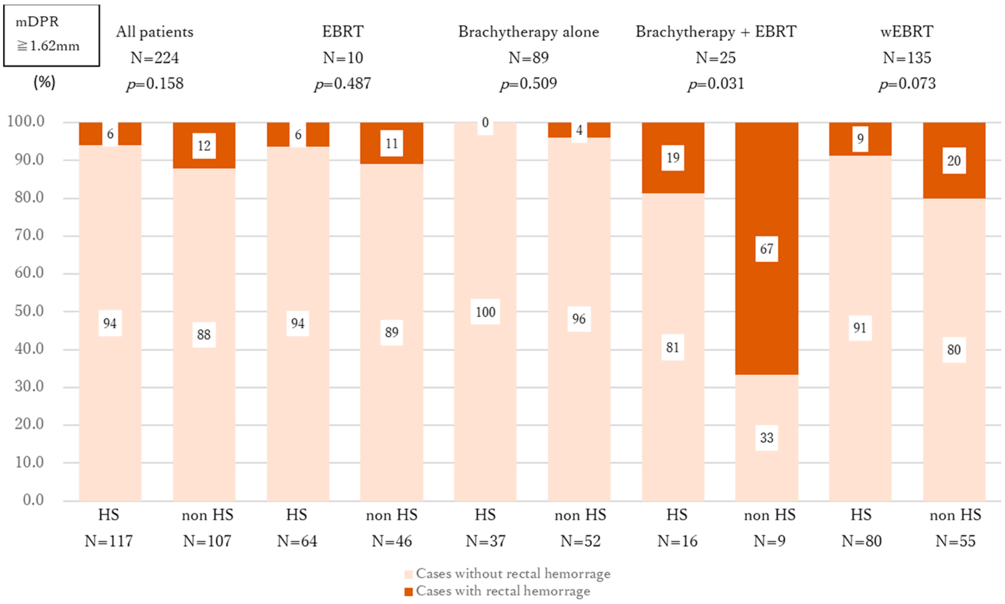


Figure S2: Effect of HS placement on the incidence of rectal hemorrhage among patients with mDPR≧1.62 mm.
